# Discovery of a magnetic conductive interface in PbZr_0.2_Ti_0.8_O_3_ /SrTiO_3_ heterostructures

**DOI:** 10.1038/s41467-018-02914-9

**Published:** 2018-02-15

**Authors:** Yi Zhang, Lin Xie, Jeongwoo Kim, Alex Stern, Hui Wang, Kui Zhang, Xingxu Yan, Linze Li, Henry Liu, Gejian Zhao, Hang Chi, Chaitanya Gadre, Qiyin Lin, Yichun Zhou, Ctirad Uher, Tingyong Chen, Ying-Hao Chu, Jing Xia, Ruqian Wu, Xiaoqing Pan

**Affiliations:** 1 Department of Chemical Engineering and Materials Science, University of California, Irvine, CA 92697 USA; 2 National Laboratory of Solid State Microstructures and College of Engineering and Applied Sciences, Nanjing University, Nanjing, Jiangsu 210093 China; 3 Department of Physics and Astronomy, University of California, Irvine, CA 92697 USA; 4 Department of Materials Science and Engineering, National Chiao Tung University, HsinChu, 30010 Taiwan; 5 Department of Physics, Arizona State University, Tempe, Arizona 85287 USA; 6 Department of Physics, University of Michigan, Ann Arbor, MI 48109 USA; 7 Irvine Materials Research Institute, University of California, Irvine, CA 92697 USA; 8 School of Materials Science and Engineering, Xiangtan University, Xiangtan, 411105 Hunan China

## Abstract

Emergent physical properties often arise at interfaces of complex oxide heterostructures due to the interplay between various degrees of freedom, especially those with polar discontinuities. It is desirable to explore if these structures may generate pure and controllable spin currents, which are needed to attain unmatched performance and energy efficiency in the next-generation spintronic devices. Here we report the emergence of a spin-polarized two-dimensional electron gas (SP-2DEG) at the interface of two insulators, SrTiO_3_ and PbZr_0.2_Ti_0.8_O_3_. This SP-2DEG is strongly localized at the interfacial Ti atoms, due to the interplay between Coulomb interaction and band bending, and can be tuned by the ferroelectric polarization. Our findings open a door for engineering ferroelectric/insulator interfaces to create tunable ferroic orders for magnetoelectric device applications and provide opportunities for designing multiferroic materials in heterostructures.

## Abstract (web summary)

Two-dimensional electron gases that form in some complex oxide heterostructures may have useful functional behavior due to the interaction of the parent materials. Here the authors show that PZT/STO interfaces can host a spin-polarized electron gas, even though the bulk materials are nonmagnetic.

## Introduction

Controllability of electronic and magnetic properties of functional materials is the cornerstone for the development of next-generation devices^1–4^. To achieve higher energy efficiency and better performance, a large wave of interdisciplinary explorations has been inspired to integrate multiple physical properties into one system. While the inherent chemical contraindication limits the fabricability of many bulk materials, heterointerfaces and multilayer films have attracted increasing attention and a multitude of intriguing physical properties have been discovered^5^. The emergent phenomena, such as the presence of high-mobility electron gas^6^, quantum Hall effect^7^, superconductivity^8,9^, ferromagnetism^10^, polarization enhancement^11,12^, and metal-insulator transition^13^, have greatly enriched fundamental science and provide platforms for technological innovations. The coexistence of ferromagnetism, superconductivity, and conducting two-dimensional electron gas (2DEG) at the interface between two insulators, e.g., LaAlO_3_ and SrTiO_3_ (LAO/STO), are among the most exciting discoveries in this realm and a number of opportunities may appear on the horizon^6,8–10,14^. In particular, ferroic-based heterostructures are intensely studied in modern materials physics and nanoscience for the realization of novel properties, since the electron spin, electric dipole and lattice strain in these materials can be continuously manipulated by magnetic, electric, and stress fields^5,15,16^.

In this work, we report the finding of emergent spin-polarized 2DEGs (SP-2DEG) at the interface between two well-known non-magnetic insulators: SrTiO_3_ (STO) and ferroelectric PbZr_0.2_Ti_0.8_O_3_ (PZT) by combination of in situ transmission electron microscopy (TEM), electrical transport measurement, magneto-optical Kerr effect (MOKE), spin polarization detection, and first-principles calculations. This adds a successful case of getting desirable physical properties from ferroelectric/insulator heterostructures and leads a different perspective for the spin-based technologies.

## Results

### Structural characterization and in situ domain switching

**Fig. 1:**
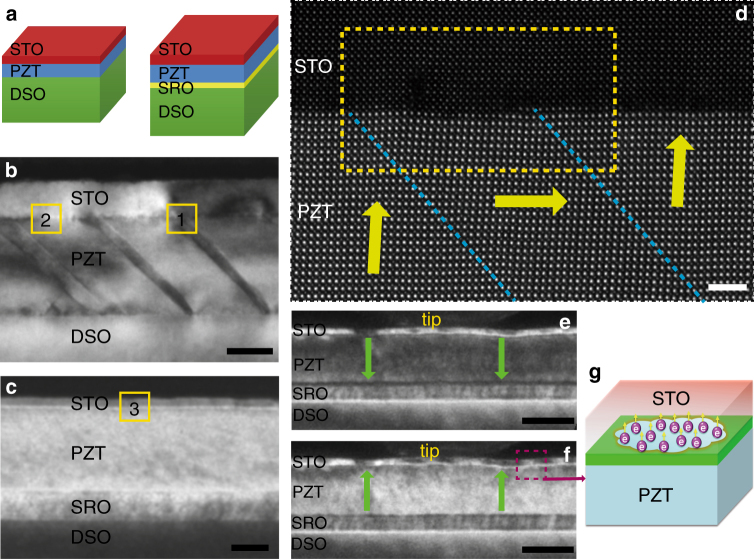
Structural characterization and in situ domain switching. **a** Schematics of the STO/PZT/DSO (left) and STO/PZT/SRO/DSO (right) heterostructures.** b** Low-magnification TEM image shows repeating 90° domain walls in the PZT layer in a STO (20 nm)/PZT (50 nm)/DSO sample. Scale bar, 20 nm. **c** Low-magnification TEM image of STO (5 nm)/PZT (50 nm)/SRO(20 nm)/DSO sample. The PZT layer is single-domain with SRO as the bottom electrode. Scale bar, 20 nm. **d** HAADF STEM image shows a subarea on the STO/PZT interface marked in the box number 1 in **b**). The 90° domain wall highlighted by the blue dashed lines. The green arrows indicate the polarization directions determined by the lead displacement vectors D_Pb_. Capacitor like domain switching in STO/PZT/SRO/DSO structure by in situ TEM. Capacitor like domain switching in STO/PZT/SRO/DSO structure by in situ TEM. Scale bar, 2 nm. **e** Dark-field TEM image shows a single-domain structure (downward polarization) at the initial state. Scale bar, 50 nm. **f** Capacitor-like domain switching occurs when a 0 →−6 V DC voltage is applied by tungsten tip. Scale bar, 50 nm. **g** The sketch of SP-2DEG induced at the interface of STO/PZT

Using pulsed laser deposition (PLD), the STO/PZT heterostructures were grown on the (110) DyScO_3_ (DSO) substrate. While the thickness of PZT layer was kept fixed at 50 nm, the thickness of the STO layer was allowed to vary (*d*_STO_ = 5, 10, 20 nm), as schematically shown in the left panel of Fig. 1a. The crystalline quality of the heterostructures was analyzed by high-resolution X-ray diffraction (XRD), atomic force microscopy, and transmission electron microscopy (TEM). We found that both STO and PZT thin films are epitaxially grown on DSO substrate and their surfaces are atomically sharp (Supplementary Figures 1, 2 and Supplementary Note 1). More structural details can be found from a dark-field TEM image of a STO (20 nm)/PZT/DSO thin film in Fig. 1b, which exhibits a mixture of *a*/*c*-domains in PZT with single crystalline epitaxy^16^. The atomic structure at the interface was further characterized by scanning TEM (STEM) and an atomic-resolution STEM high-angle annular dark-field (HAADF) image is shown in Fig. 1d. The STO film is coherent with PZT without noticeable defects, except for one dislocation near the 90° domain. The polarization mapping shows an upward ferroelectric polarization in the *c*-domain of PZT, pointing toward STO in the STO/PZT/DSO structure^17^ (Supplementary Figure 3a, b). Interestingly, the PZT domain structure can be substantially altered by adding an epitaxial SrRuO_3_ (SRO) bottom electrode as schematically shown in the right panel of Fig. 1a. In the STO/PZT/SRO/DSO film, PZT becomes mono-domain (Fig. 1c) with its electric polarization pointing towards SRO^15,18^ (Supplementary Figure 3c, d). Furthermore, the polarization of PZT can be switched to upward by applying a negative bias on top of the STO layer, as depicted in Fig. 1e, f. One important finding here is that almost the entire domain abruptly switches when the negative bias reaches −5 V (Fig. 1f), much different from the way of domain switching for an uncapped PZT layer for which the switching starts from a small area near the tip and gradually expands as the bias increases^15^ (Supplementary Movies 1 and Figure 4). This uniform domain switching behavior is similar to what occurs in a planar ferroelectric capacitor (Supplementary Movies 2) and suggests a conducting STO/PZT interface, which becomes a top electrode and thus a homogeneous electric field is generated across the PZT film when the bias is applied, as illustrated in the Fig. 1g.

### Transport measurements

**Fig. 2:**
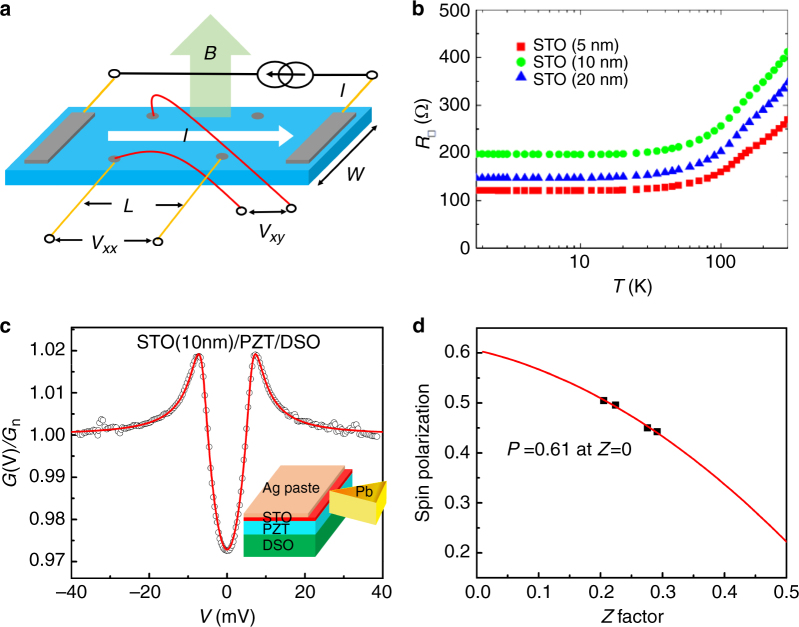
Transport measurements. **a** Schematics of the Hall-bar configuration. **b** Sheet resistance of the STO/PZT/SRO/DSO thin films with various STO thicknesses at a temperature range of 1.8–300 K. Andreev spectra measurement in STO/PZT/DSO heterostructure at the temperature of 1.5 K. **c** Representative Andreev spectra (open circles) of a point contact between Pb superconductor and STO/PZT interface with the best fits to the modified BTK model (solid curves), where *T* = 1.51 K, *P* *=* 0.50, *Δ* *=* 1.31 meV, *r*_E_ = 3.76, *Z* *=* 0.21, and *R* = 899 Ω. The inset is the schematic of point contact established at STO/PZT interface. **d** The dependence of spin polarization (*P*) values as a function of the interfacial scattering *Z* factor in STO/PZT/DSO heterostructure

To validate this hypothesis, we examined three STO/PZT/SRO/DSO samples with different STO thicknesses through Hall-effect measurements over a temperature range of 1.8–300 K, with the experimental geometry sketched in Fig. 2a. Significantly, the sheet resistance *R*_□_ becomes independent of *T* (Fig. 2b) below 20 K, suggesting metallic characteristics of these samples. Note that *R*_□_ varies within a small range from 120 to 200 Ω for the sample with different STO thicknesses, most likely due to the inevitable misalignment of the electrical contacts. The weak thickness dependence is a clear indication that the STO/PZT interface becomes conducting by the formation of interfacial 2DEG. We also measured the Hall resistance (*R*_xy_) and estimated the sheet carrier concentration of about 10^13^ cm^−2^, which is a reasonable value for oxide 2DEG systems^19^ (Supplementary Figure 5). The existence of a 2DEG at the STO/PZT interface was further confirmed by conductive atomic force microscopy (cAFM). In the Supplementary Figure 6, only two parallel conductive regions were found in the cAFM image for a STO/PZT/SRO/DSO sample, which correspond to local conducting channels at the STO/PZT interface and SRO layer, respectively. Importantly, we remark that the conducting feature of the STO/PZT interface can be diminished by changing the ferroelectric polarization patterns of the underlying PZT film. If we remove the bottom SRO layer, the STO/PZT interface becomes macroscopically resistive due to the emerging 90°domains (Fig. 1b), which effectively block conducting paths and render the insulating or poor conducting state at the STO/PZT interface in a long-range^20^ (Supplementary Figure 7).

To confirm this local metallic behavior, Andreev reflection spectroscopy (ARS) was employed to study the STO/PZT interface of STO (10 nm)/PZT/DSO heterostructure^21–23^. As schematically drawn in the inset of Fig. 2c, the cross-section sample of STO/PZT/DSO is in a point contact to a superconducting Pb knife-wedge tip at 1.5 K. To reduce the effect of the 90° domain, a Ag paste was applied about 50–100 µm close to the fresh edge of the cross-section. To make sure the contact to STO/PZT interface, scratches were made on the sample below Ag paste. Over 20 contacts at different locations on the cross-section were measured and the representative ARS spectrum, as expressed by the normalized differential conductance spectra *G*(*V*)/*G*_n_, is shown in Fig. 2c with the bias voltage range of ±40 mV. The metallic behavior with a resistance (*R*) of about 1 kΩ was found for all contacts, indicating the presence of 2DEG in the sample. Very interestingly, by analyzing the spectra *G*(*V*) using the modified Blonder–Tinkham–Klapwijk (BTK) model^21–23^, we obtained a large spin polarization (*P*) value for each contact. The best fits are shown in Fig. 2c as solid curves where spin polarization (*P*), the superconducting gap (*Δ*), interfacial scattering (*Z*), and extra resistance (*r*_E_) are fitting parameters. By extrapolating *Z* to zero, an intrinsic spin polarization of 61% for STO/PZT/DSO heterostructure is obtained, strongly suggesting a ferromagnetic nature of these conduction electrons at the STO/PZT interface (Fig. 2d). Details of ARS results can be seen in Supplementary Figure 8. Obviously, the ARS results not only clearly confirm the metallic property of the STO/PZT interface, but also demonstrate that the interfacial 2DEG is spin-polarized.

### Sagnac interferometer and MOKE measurements of STO/PZT/DSO samples

**Fig. 3:**
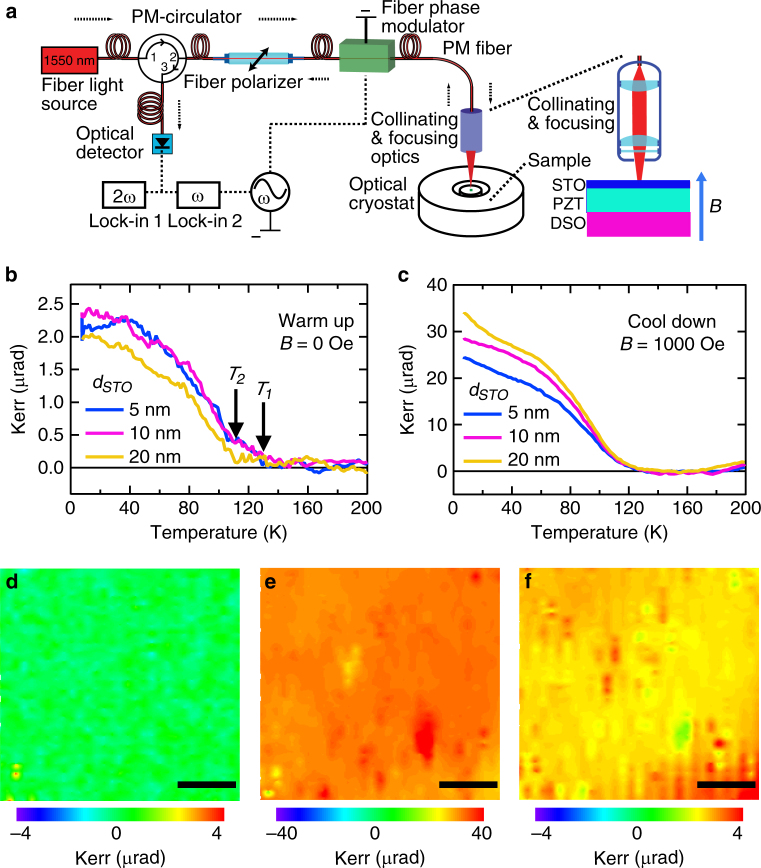
Sagnac interferometer and MOKE measurements. **a** Schematics of the all-fiber Sagnac interferometer. **b** Kerr effect during 0 Oe warmup and (**c**) 1000 Oe cool-down for three STO (5, 10, 20 nm)/PZT/DSO samples with the same 50 nm PZT thickness. **d** Kerr signal image mapping over a 60 × 60 μm^2^ scan area in the STO (5 nm)/PZT/DSO sample at 130 K. Scale bar, 15 μm. **e**, **f** Kerr signal image mapping in the same region at 4 K under 1000 Oe and 0 Oe field, respectively, both showing uniform ferromagnetism signal distribution. Scale bar, 15 μm

While transport experiments above suggest the emergence of a spin-polarized 2DEG at the STO/PZT interface^24^, the ferromagnetic property is further examined by Sagnac MOKE measurement. Loopless fiber-optic Sagnac interferometry, a surface magnetic probe technique based on the ultra-sensitive MOKE method, was employed here in order to avoid the unwanted signal from the thick paramagnetic DSO substrate^25–29^. As shown in Fig. 3a, the Kerr angle *θ*_K_ was detected by measuring the optical phase difference between counter propagating oppositely circularly polarized light waves reflected from the sample surface. This method has an unprecedented 0.01 μrad resolution and avoids artifacts such as linear birefringence commonly seen in samples with anisotropic strain^28,29^. During the measurements, the samples were first cooled to 4 K with a 1000 Oe magnetic field applied perpendicular to the sample, and then warmed up in 0 Oe. During the 0 Oe warmup (Fig. 3b), *θ*_K_ ≈ 2.0 *μ*rad was observed at 4 K, and it gradually dropped to zero at the critical temperature *T*_c_, which is 110 K (*T*_2_) for *d*_STO = _20 nm and 130 K (*T*_1_) for *d*_STO_  =  5 and 10 nm. The spontaneous Kerr signal at zero magnetic field suggests a ferromagnetic phase transition at *T*_c_ for the STO/PZT heterostructures. At low temperature, values of *θ*_K_ (≈2.0 *μ*rad) for three different STO thicknesses are almost identical. Furthermore, no Kerr signal was observed within 0.05 μrad uncertainty during similar zero-field warmup measurements in either a bare DSO substrate^27^, a PZT (50 nm)/DSO or STO (10 nm)/DSO thin film (Supplementary Figure 9). Moreover, HRXRD analysis and STEM images show that the same quality of STO layer on PZT and DSO substrates (Supplementary Figure 10). Therefore, the observed ferromagnetism must originate from the STO/PZT interface. During the cooldown in the 1000 Oe field, we expect a contribution to *θ*_K_ from the thick paramagnetic DSO substrate. This offset was found to be ~5 μrad and was subtracted from Fig. 3c. Again, *θ*_K_ at 4 K is basically *d*_STO_-independent, confirming that the ferromagnetism occurs at the STO/PZT interface even in a magnetic field. From the 10 times enhancement of Kerr rotation compared to its zero-field counterpart, we estimated that the magnetic easy axis is close to the in-plane direction, with a tilting angle of about 2 degrees. Furthermore, the saturated magnetization of 6.6 × 10^12^ μ_B_ cm^−2^ is close to the value previously reported for the LAO/STO system^14^.

Unlike the interfacial ferromagnetism observed in LAO/STO or LaMnO_3_/STO in the form of isolated micrometer magnetic patches^9,30^, the ferromagnetism at our STO/PZT interface is rather uniform. The Kerr signal acquired at 130 K (above *T*_c_) on a STO (5 nm)/PZT/DSO sample is zero over a 60 × 60 μm^2^ scan area (Fig. 3d) with an approximate pixel size of 1 μm^2^. At 4 K, the image mapping (Fig. 3e) taken in the same region under a 1000 Oe field shows a uniform distribution of the Kerr signal around 30 μrad. Although the Kerr signal is reduced to 2 and 3 μrad in zero-field, the magnetization is still even in the entire region of our observation (Fig. 3f). The uniform ferromagnetism, along with the structural characterization and the results of electrical transport measurement discussed above, clearly indicate that the spin-polarized 2DEG (SP-2DEG) is not due to crystal misfits or defects, but caused by a different mechanism.

## Discussion

**Fig. 4:**
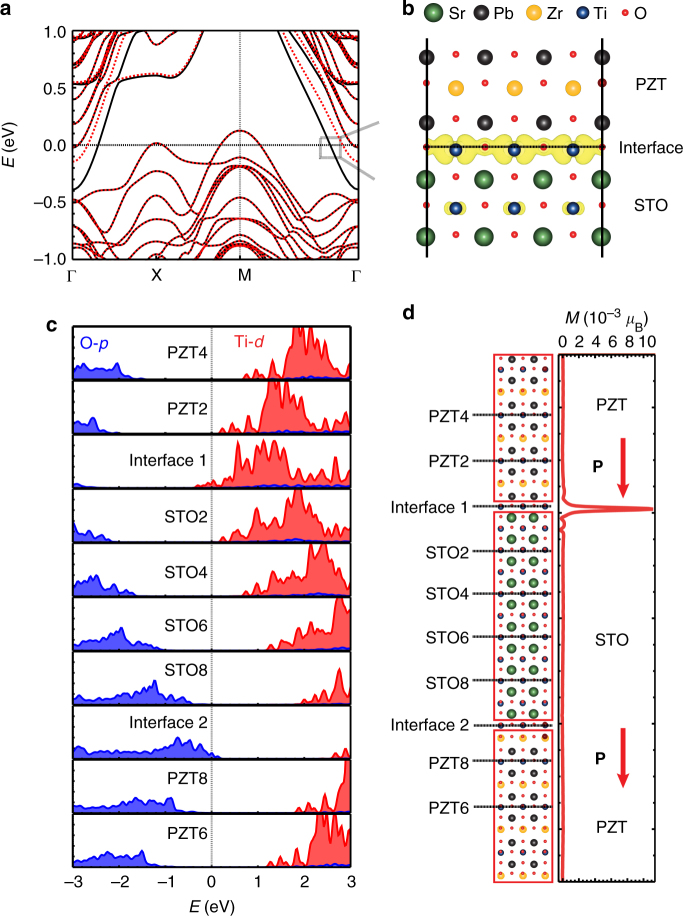
Theoretical calculations.** a** Calculated band structure of the STO/PZT heterostructure with *U*_eff_ =  2.3 eV for Ti and Zr *d*-orbitals. The majority-spin and minority-spin bands are represented by black solid and red dashed lines, respectively. **b** The squared wave-function of the state that crosses the Fermi level. **c** Atom-resolved DOS of the STO/PZT heterostructure. The vertical dashed line at *E* = 0 represents the position of the Fermi level. Blue and red colors denote O-2*p* and Ti-3*d* contributions, respectively. **d** The planar-average of spin density of the STO/PZT heterostructure along the polarization (**P**) axis. The corresponding atomic structure is shown in the left side

To understand the origin of the emergent SP-2DEG in STO/PZT films, first-principles calculations were carried out using the Vienna ab initio simulation package (VASP)^31^. We employed a supercell consisting of alternatively stacked STO and PZT layers along the *c*-axis. Interestingly, spin-polarized metallic states appear at the interface between STO and PZT layers even though both are insulators, as seen from the two bands crossing the Fermi level and their wave function features in Fig. 4a and b. The curves of the atom-resolved density of states (DOS) for different O and Ti atoms show that the conduction and valence bands are almost linearly shifted along the *c*-axis due to the internal electric field produced by the polarization in the ferroelectric PZT layers (Fig. 4c). Obviously, the band bending is the main driving force for the formation of 2DEG as states of the interfacial atoms are pushed across the Fermi level. Since the conduction bands of STO mainly consist of Ti-3*d* orbitals, the emergence of magnetization is understandable according to the Stoner model. Furthermore, the inclusion of a small Hubbard *U* correction for Ti-3*d* orbitals in DFT calculations increases the energy gain of magnetization (Supplementary Figure 11). The size of the magnetic moment, 0.02–0.05 μ_B_ per cell or (1.2–3.6) × 10^13^ μ_B_ cm^−2^ is somewhat larger than our estimation from the MOKE measurement, 6.6 × 10^12^ *μ*_B_ cm^−2^. Furthermore, the population ratio of the majority spin and minority spin electrons is about 4:1 as estimated from the wave vectors where the two parabolic bands intercept the Fermi level. This corresponds to a spin polarization of 60%, also in good agreement with the ARS results. Therefore, the SP-2DEG observed experimentally can be attributed to the magnetized interface states that result from the interplay between band bending and local Coulomb interaction at the interfacial Ti sites.

Note that we have two STO/PZT interfaces in our theoretical calculation, whereas only one of them exists in our experimental samples. An intriguing explanation is that although both interfaces are metallic, mediated by the 2DEG at Interface 1 and 2D hole gas (2DHGs) at Interface 2, magnetization only arises at the interface where the electric polarization of PZT is pointing towards STO (Interface 1 in Fig. 4d). From the DOS features in Fig. 4c, one can see that the 2DHGs at Interface 2 has features of O-2*p* orbitals as a result of the upward band bending. Our calculations indicate that Interface 1 is slightly more favorable than Interface 2 (Supplementary Figure 12), so the electric polarization in PZT should be more likely to point toward STO in samples. Furthermore, one may manipulate the magnetic state of the 'STO/PZT interface with a bias, as done in Fig. 1e, f. This adds a knob to tune the magnetic state of oxide films, as desired for multiferroic materials. Finally, our DFT calculations indicate that the STO/PZT interface with 90° domain is insulating and non-magnetic (Supplementary Figure 13), in line with experimental observations. This is understandable since no band bending occurs in STO when the polarization in PZT points sideways.

Note that ferromagnetism and 2DEG were also reported for LAO/STO heterostructure, but results from different groups appeared to be inconsistent^32–37^. It was proposed that oxygen vacancies play an important role for the presence of conductivity and ferromagnetism^33,34,37^. To see if this mechanism is also applicable for the STO/PZT interface, electron energy-loss spectroscopy (EELS) was applied to examine oxygen vacancies. As is clearly shown in EELS (see Supplementary Figure 14), no obvious evidence of oxygen vacancies could be found around the STO/PZT interface. Furthermore, Sagnac interferometer measurement for Kerr rotation angle barely changes after the sample is annealed in oxygen (see Supplementary Figure 15). Therefore, we are convinced that oxygen vacancy is not the source of ferromagnetic 2DEG observed at the STO/PZT interface, different from that of LAO/STO system^34^. EELS of Ti-L_2,3_ edges were acquired as a function of the distance from the STO/PZT interface as well (see Supplementary Figure 16). It is well known that in EELS the Ti-*L*_2,3_ edge shows well-defined four peaks in compounds in which Ti^4+^ is present, such as STO, but consists of two broadened peaks in the presence of Ti^3+^. Our EELS results show that the characteristic four peaks of the Ti-*L*_2,3_ edge are seen in STO far away from the interface, but tend to form two broad peaks at the interface. The broadening feature is attributed to the presence of Ti^3+^ near the STO/PZT interface. Moreover, the analysis by X-ray photoemission spectroscopy (XPS) shows that the spectrum of the bulk phase of STO layer in STO/PZT heterostructure is identical to the standard STO reference (Supplementary Figure 17 and Note 2). Therefore, we believe that this Ti^3+^ introduced by a ferroelectric polar gating effect at the STO/PZT interface contributes to the conductivity and ferromagnetism^8,36–39^.

In summary, a SP-2DEG with uniform magnetization and high conductivity was discovered at the interface between perovskite STO and ferroelectric PZT, using TEM, electrical transport, and MOKE measurements. Quantitatively, the spin polarization value of this 2DEG at STO/PZT interface is determined to be about 61% by Andreev reflection spectroscopy. Theoretical calculations revealed that the origin is the band bending in STO layers and the possibility of switching the magnetic states via electric bias. The coexistence of high conductivity and a switchable magnetic state at the interface between two non-magnetic insulators is reported for the first time. The present work demonstrates a prototypical case of designing interface physics from the interplay of multiple factors in ferroelectric-based heterostructures. It is foreseeable that these results and concepts are useful for the search and design of heterogeneous multiferroic materials and devices^40–42^.

## Methods

### Materials system

The materials used in this study are lead zirconate titanate Pb(Zr_0.2_Ti_0.8_)O_3_ (PZT), strontium titanate (SrTiO_3_), strontium ruthenate (SrRuO_3_) and dysprosium scandate (DyScO_3_). PZT is a classical ferroelectric at room temperature^43^. STO is an important multifunctional material in oxide-based electronic devices for its superior thermal and chemical stability and excellent lattice match with other functional oxides^44–46^. In its bulk form, STO is a wide bandgap insulator and is non-magnetic^47,48^. SRO is well known as a ferromagnetic oxide metal. It exhibits a transition to a ferromagnetic state at *T*_c_~160 K. At room temperature, it shows a “bad metal” behaviors^49^. DSO is a paramagnetic insulator, which shows a magnetic phase transition at 3.1 K^50^.

### Thin film growth

SrTiO_3_ layers with different thicknesses (5, 10, 20 nm) were deposited on tetragonal Pb(Zr_0.2_Ti_0.8_)O_3_ (PZT) oriented in the [001] direction with a thickness of 50 nm, grown on (110) DyScO_3_ substrates with/without a 20 nm SrRuO_3_ buffer layer. An oxygen pressure of 100 mTorr was maintained during the growth of PZT and STO at 650 °C. After growth, samples were cooled to room temperature under an oxygen partial pressure of 200 Torr to minimize oxygen vacancies in the film, especially to eliminate potential current leakage through PZT^51^.

### TEM and in situ experiments

Cross-sectional TEM samples were prepared following the mechanical thinning, polishing and ion milling process. The film was glued to a sacrificial silicon wafer in a cross-sectional configuration and then polished to a thickness of ~20–30 μm in wedge shape using a tripod polisher. After mechanical thinning and polishing, the sample was further ion-milled to electron transparency in a Gatan Precision Ion Polishing System 2, with the ion gun voltage of 4 kV and a milling angle of ±4°. At the final step of ion milling, a 0.1 kV voltage was used to mildly mill the sample and remove the surface amorphous layer.

STEM experiments were carried out in a FEI Titan 80–300 microscope at Nanjing University. The microscope is equipped with a monochromator and double aberration correctors. At an accelerating voltage of 300 kV, the resolution in STEM mode is ~0.6 Å. The accelerating voltage, the convergence angle of the incident electrons and the collection angle for STEM HAADF imaging are 300 kV, 22 mrad and 79–200 mrad, respectively.

In situ experiments were carried out using a Nanofactory scanning probe microscopy platform for TEM^52^. Electrical bias was applied between an electrochemically etched tungsten tip, which was used as a movable surface electrode, and the conductive buffer layer SRO bottom electrode, which was connected to the holder ground using silver paint. External voltages were applied using an HP 6614c for DC voltages. The domain structure was imaged in real-time in the dark-field imaging mode. The real-time movies (Supplementary Movies 1 and 2) were recorded by a Gatan TV-rate camera.

### Polarization mapping

The atomic displacement **D**_Pb_, which is responsible for the spontaneous polarization **P**_S_, is directly measured from the STEM HAADF image by fitting the image with two-dimensional (2D) Gaussian functions. After fitting, the atomic positions of both *A*-site and *B*-site atoms are extracted from the centers of these 2D Gaussian functions. **D**_Pb_ is defined as the off-center displacement of the corner *A*-site Pb atoms with respect to the center of four surrounding *B*-site Zr/Ti atoms^17,53^.

### Transport measurements

Low-temperature transport property measurements were carried out at University of Michigan over the temperature range of 1.8–300 K in a Quantum Design Magnetic Property Measurement System (MPMS) equipped with a 5.5 T magnet, using a Linear Research ac bridge with 16 Hz excitation. All samples were cut to a width-length ratio of ~1:2. Hall-bar-shaped device with six fine indium contacts was carefully prepared to reach STO/PZT interface, and no contact metal touches the edges or sides of the sample or SRO layer (as schematically shown in Fig. 2a). The uncertainty of electrical resistivity and Hall coefficient is estimated to be lower than ± 5%.

### Point contact Andreev reflection spectroscopy

The transport through an interface between a normal metal and a superconductor is dominated by a process called Andreev reflection^21,22,54^. When the applied voltage is less than the superconductor (SC) gap *Δ* (*V* <* Δ*/e) at normal metal/SC interface, a current is injected from a normal metal into a SC and this normal current must be converted into a supercurrent. For each injected electron from the normal metal, it must be accompanied by another electron with proper spin to form a Cooper pair to propagate in the SC, consequently reflecting a hole back into the normal metal, which is equivalent to doubling the conductance as compared to the normal state conductance (*G*_n_ at *V* >> *Δ*/e). This process is so-called Andreev reflection. Different from a normal metal, there is an imbalance in the number of spin-up and spin-down at the Fermi level in Ferromagnetic-metal. Therefore, the Andreev reflection probability is limited by the minority carriers and the conductance is suppressed at ferromagnetic-metal/SC interface. Thus, the conductance at metal/superconductor interface can be used to determine the spin polarization (*P*) value of any metal. The measurements of differential conductance d*I*/d*V* = *G*(*V*), with *I* the current, *V* the bias voltage across the contact, and *G* the conductance, were carried out by varying *V* in a cryo-station. In this work, a point contact is established using a superconducting Pb knife edge on a cross-section of the STO/PZT/DSO structures at 1.5 K. Then, the collected ARS is analyzed within the framework of Blonder-Tinkham-Klapwijk (BTK) theory. By analyzing the spectra *G*(*V*) using the BTK model, the spin polarization *P* value of the conductive STO/PZT interface can be determined.

### Sample preparation for cAFM characterization

Conducting atomic force microscope (cAFM) is a powerful technique to map the spatial distribution of conductivity in complex heterostructure^55,56^. To provide direct experimental evidence into the origin and nature of electrical conductivity in these heterostructures, we addressed this issue by probing local conductivity using cAFM in cross-section geometry. For cAFM, measurements were carried out using an NT-MDT Spectra scanning probe microscope. Conductive diamond coated probes with a nominal force constant 16 N m^−1^ were used for all scans presented in this paper. Conductance maps were collected with a sample bias of 1 V while the tip was held at ground. The sample preparation for cross-sectional scanning probe microscopy (SPM) measurements was the same as the standard procedure for TEM specimens. The film and electrodes were connected to the back contact by silver paint.

### Sagnac MOKE measurement and imaging

Figure 3a shows a schematic of the Sagnac interferometer based scanning microscope. In this fiber-optic Sagnac interferometer, circularly polarized lights at 1550 nm wavelength with opposite chirality and opposite momentum are reflected from the same spot on the sample surface through a N.A. = 0.65 focusing lens. The phase difference between these two light, being time reversal of each other, could only originate from time-reversal-symmetry effects, such as magnetism. As a result, artifacts such as optical birefringence and dichroism are rejected at a level better than 5 parts in a billion. For imaging, the fiber head and the focusing optics are mounted on a piezo stage capable of 50 nm step size in both the x- and y-directions. The spatial resolution is determined by the optical diffraction limit to about 1200 nm. During the measurements, the optical power on the sample is 0.01 mW, which is too small to induce any recognizable sample heating effect at these temperatures.

### Computational details

The first-principles calculations were performed using the projected augmented plane-wave method^57,58^ as implemented in the Vienna ab initio simulation package (VASP). The valence electron configurations for Sr, Pb, Ti, Zr, and O are 4*s*^2^4*p*^6^5*s*^2^, 6*s*^2^6*p*^2^, 3*p*^6^3*d*^2^4*s*^2^, 4*s*^2^4*p*^6^4*d*^2^5*s*^2^ and 2*s*^2^2*p*^4^, respectively. The generalized gradient approximation (GGA) of the Perdew-Burke-Ernzerhof type was used for the description of exchange-correlation interactions among electrons^31,59^. A 10 × 10 × 1 k-point grid was employed for STO/PZT heterostructure containing 10-unit cell STO and PZT layers stacked along the *c*-axis. Slab structures separated by ~20 Å vacuum along the surface normal were constructed to study the preferential polarization direction in the STO/PZT heterostructure. To describe the correlation effect properly, the GGA + *U* method was used for the *d*-orbitals of Ti and Zr (*U* = 3.2 eV, *J* = 0.9 eV)^59^. The energy cutoff for the plane-wave-basis expansion was set to 400 eV. Positions of all atoms are fully relaxed until the convergence of total energies becomes better than 0.1 meV.

### *U* effect on magnetization

The effect of *U* value of 3*d*-transition metals (Ti, Zr) was investigated on the magnetization of the STO/PZT heterostructure. As shown in Supplementary Figure 11a, the STO/PZT heterostructure shows a weak magnetization even if + *U* effect is not included, and the magnetic moment increases with *U*_eff_. The energy gain from spin polarization also increases for the large *U*_eff_ values. (Supplementary Figure 11b). The typical *U*_eff_ value of Ti atom is in the range of 2.3–4.3 eV for STO^60^, so magnetization of 2DEG in the STO/PZT heterostructures should be robust. Furthermore, the *U* value of Zr atom does not affect the magnetic property of the STO/PZT heterostructure (Supplementary Figure 11c).

### Preferential polarization direction

In the superlattice model of STO/PZT, STO and PZT layers form two different interfaces: one has the polarization direction of PZT pointing to STO (Interface 1, magnetic), the other has the opposite direction (Interface 2, non-magnetic). Since the Interface 2 is less stable than the Interface 1, the spontaneous magnetization is very likely to appear in STO/PZT heterostructures, unless one deliberately changes the direction of polarization. It appears that the polarization in PZT affects bands of many STO layers near the Interface 1 whereas its effect is quickly reduced nears the Interface 2.

### EELS

High spatial resolution STEM-EELS experiments were performed using a Nion UltraSTEM 200 at UC, Irvine, equipped with C3/C5 corrector and high-energy resolution monochromated EELS system (HERMES). The instrument was operated at 100 kV with convergence semi-angle of 30 mrad and with a beam current of ~100 pA. A dispersion of 0.75 eV per channel was used, and the dwell time was 1 s per pixel. The background in each spectrum was removed by power-law function in commercial software package DigitalMicrograph.

### High-resolution X-ray diffraction

The film structures were examined at room temperature by HRXRD using a Rigaku Smartlab diffractometer equipped with a Cu K_α1_ source of radiation (*λ* = 0.15406 nm) and a Ge (220 × 2) monochromator. The 2*θ*/*ω* XRD patterns along (*00* *L*) crystallographic axis, symmetrical reciprocal space mappings (RSM) around DSO (*002*) and asymmetrical RSM around DSO {103} were measured.

### X-ray photoemission spectroscopy

XPS experiments were performed using a Kratos axis ultra X-ray photoelectron spectrometer, which uses monochromated Al Kα radiation as the X-ray source and the total energy resolution is ~0.5 eV. All spectra were acquired at a normal takeoff angle at room temperature.

### Data availability

The data that support the findings of this study are available from the corresponding authors on request.

## Electronic supplementary material

Supplementary Information

Description of Additional Supplementary Files

Supplementary Movie 1

Supplementary Movie 2
