# Supplementary material for: Discovery of a magnetic conductive interface in PbZr0.2Ti0.8O3 /SrTiO3 heterostructures
Source: Nat Commun. 2018 Feb 15;9:685. doi: 10.1038/s41467-018-02914-9 (PMC5814552; doi:10.1038/s41467-018-02914-9)
Supplement: Supplementary file 1 — Supplementary Information [file 41467_2018_2914_MOESM1_ESM.pdf]

Supplementary Figures

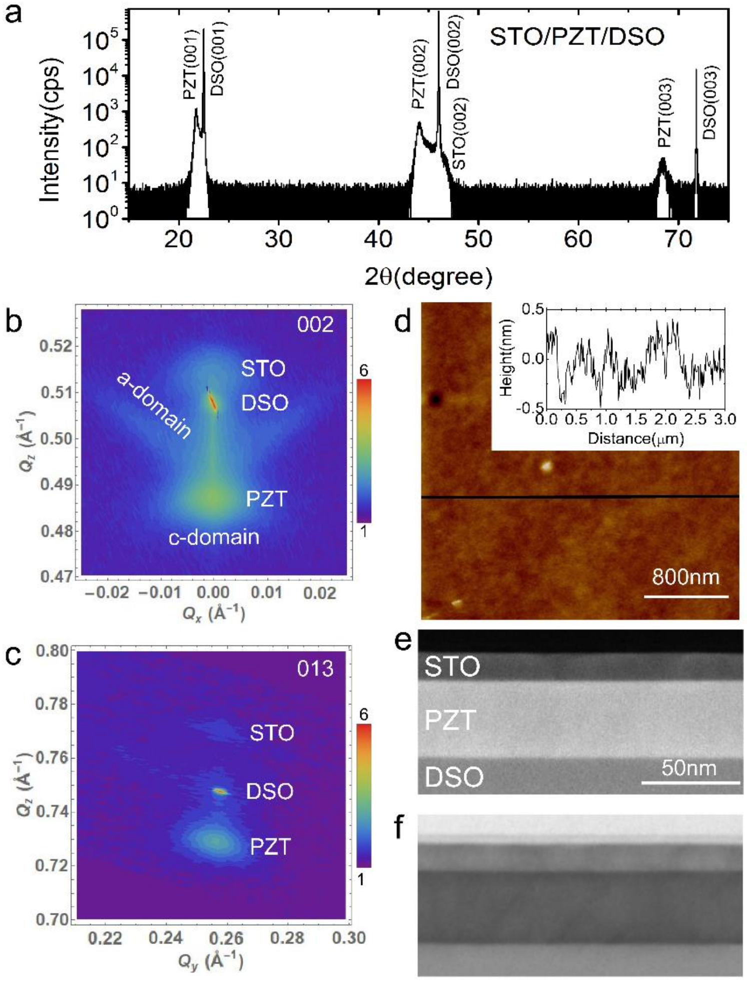

Supplementary Figure 1 | Structural characterization of the STO/PZT/DSO. (a) Out-of-plane HRXRD 2θ-ω scans in STO/PZT/DSO along film growth direction. (b) High-resolution reciprocal space map around the DSO (002) reflection. (c) Asymmetrical reciprocal space map around the DSO (013) reflection, confirming the epitaxial growth. The color bar presents the intensity level in log scale. Q_z and Q_x are the components of the reciprocal space vector along the [00l] and [hh0] directions, respectively. (d) Surface morphology of the (001) epitaxially grown STO (10 nm)/PZT/DSO measured by AFM in 3×3 μm^2 area. Low magnification HAADF STEM image: HAADF (e) and bright field image (f).

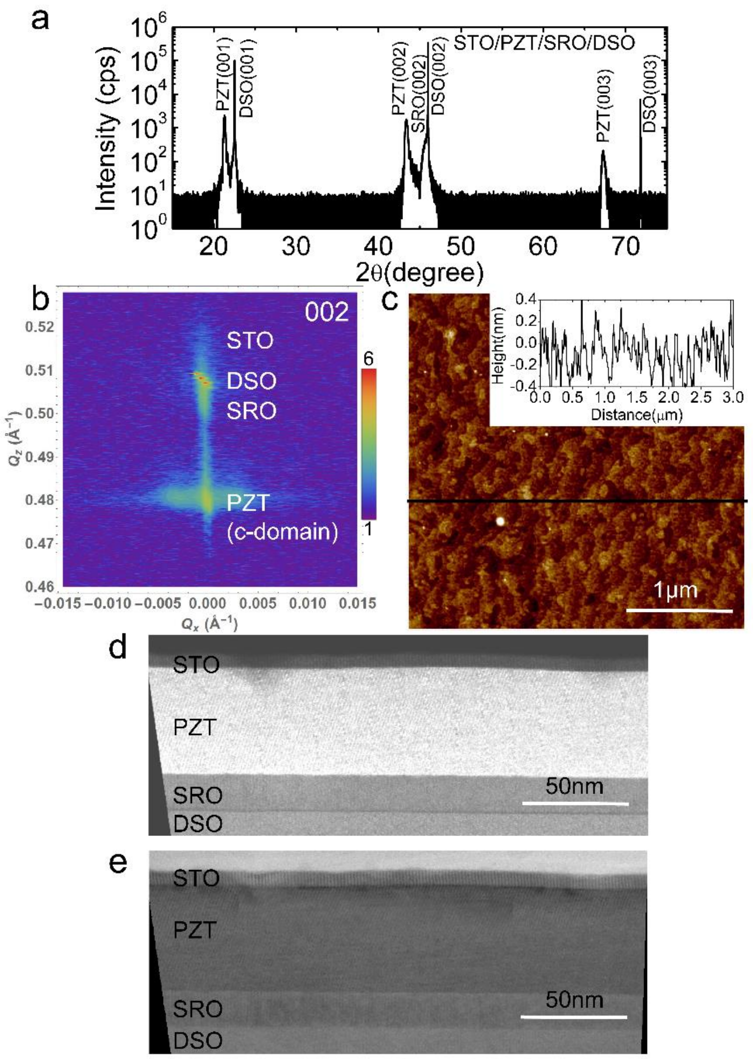

Supplementary Figure 2 | Structural characterization of the STO/PZT/SRO/DSO. (a) Out-of-plane HRXRD 2θ-ω scans in STO/PZT/SRO/DSO. (b) High-resolution reciprocal space map around the DSO (002) reflection. (c) Surface morphology of the (001) epitaxially grown STO (10nm)/PZT/SRO/DSO measured by AFM in 3×3 μm^2 area. Low magnification HAADF STEM image: HAADF (d) and bright field image (e).

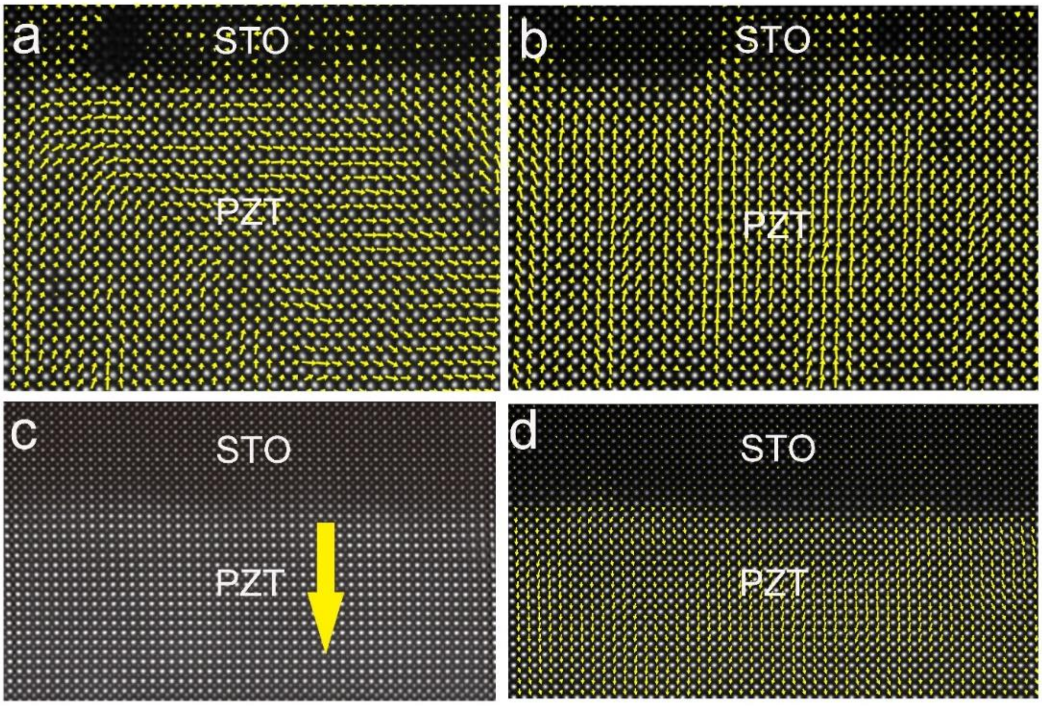

Supplementary Figure 3 | Polarization mapping around the STO/PZT interface. (a) Polarization vector map of STO/PZT interface around 90° domain area in STO/PZT/DSO sample, corresponding to the rectangle box number 1 in Fig. 1b. (b) Polarization vector map of STO/PZT interface corresponding to the rectangle box number 2 in Fig. 1b. (c) HAADF STEM image shows the sharp STO/PZT interface in STO/PZT/SRO/DSO sample, corresponding to the rectangle box number 3 in Fig. 1c, where the PZT layer is dominantly single-domain with polarization pointing away from STO layer, as marked by the arrow. (d) Polarization vector map of STO/PZT interface in (c).

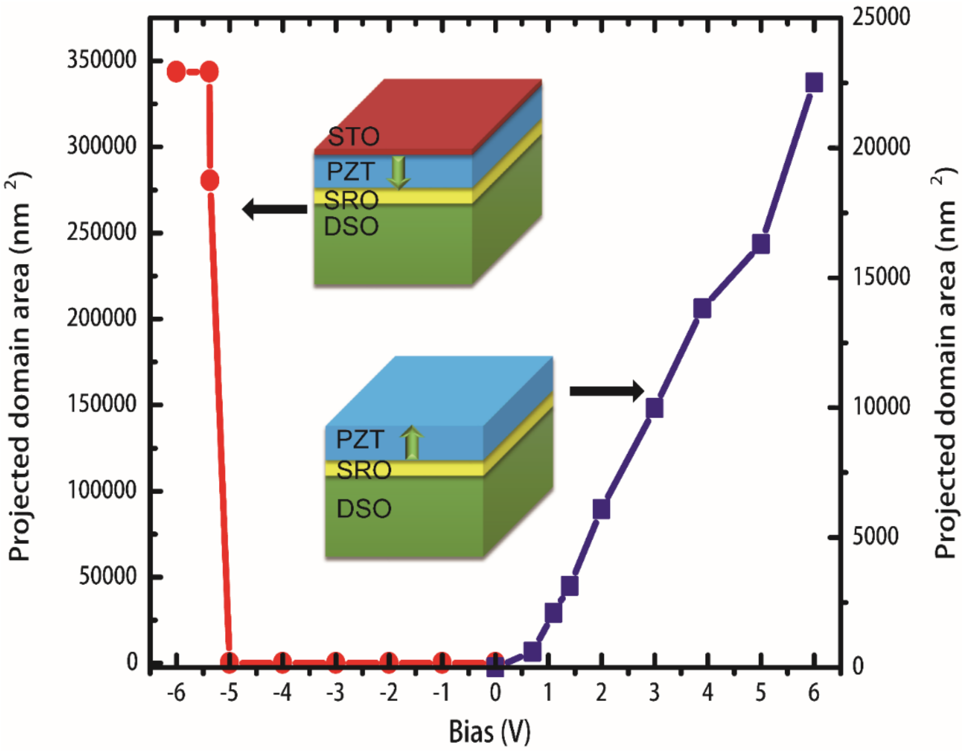

Supplementary Figure 4 | *In-situ* domain switching in the STO/PZT/SRO/DSO. The switched domain area as a function of bias extracted from Supplementary Movies 1 and 2 for STO/PZT/SRO/DSO (red dot) and PZT/SRO/DSO (blue rectangular). The schematics of the corresponding multilayer structures are shown in the inset. The initial polarization directions of PZT layer are marked by arrows.

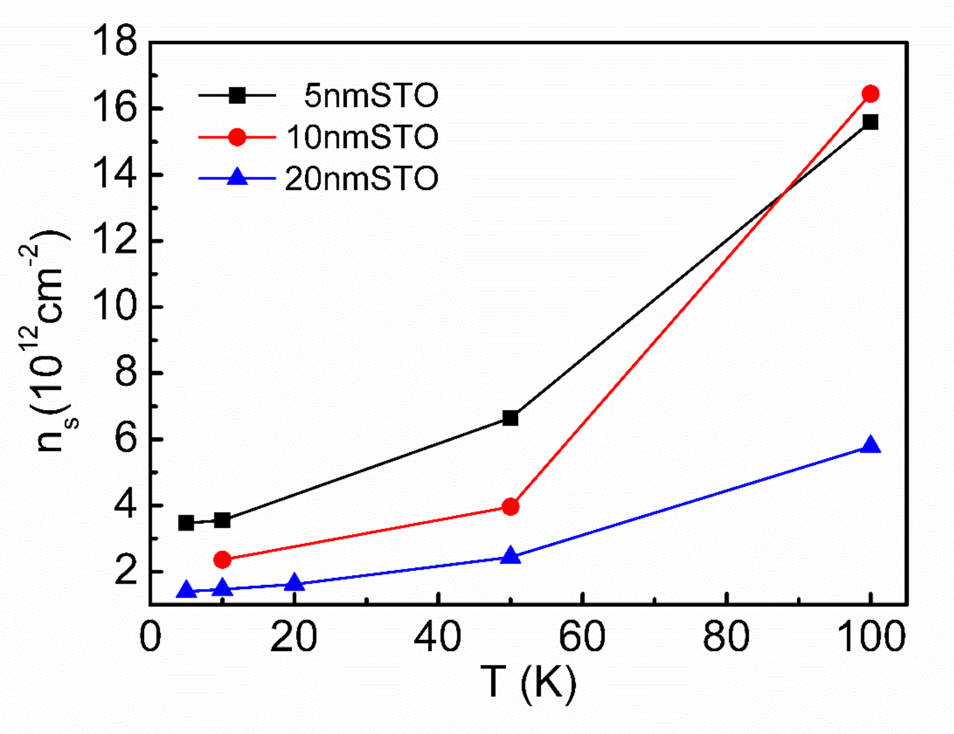

Supplementary Figure 5 | Electrical transport properties. Nominal sheet carrier concentration in three STO/PZT (50 nm)/SRO (20 nm)/DSO films with different STO layer thickness. Measured temperature range is 5-100 K. Hall carrier concentrations were calculated according to $n_s=-1/(eR_h)$, where e is electron charge and R_h is Hall coefficient.

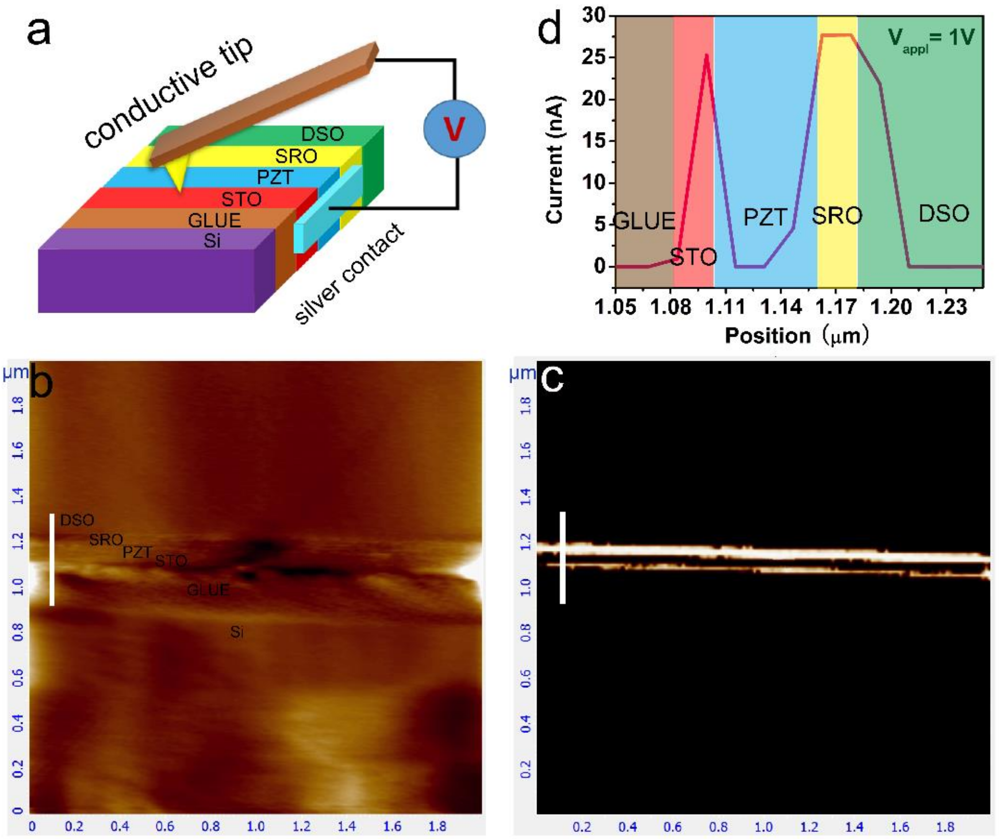

Supplementary Figure 6 | Conductive AFM measurement. (a) Schematics of the heterostructure. The STO (20 nm)/PZT (50 nm)/SRO (20 nm)/DSO sample was glued to a Si wafer and prepared into a TEM specimen. (b) Topography map (contact mode) of the specimen. Scan area is 2×2 μm^2. (c) Current map on the same scan area (indicated by corresponding bars in b) and c) obtained simultaneously while applying a 1 V bias. Dark areas are resistive. The thick bright line is the SRO layer, and the thinner line correspond to the STO/PZT interface. (d) Recorded current as the AFM tip swept across the specimen. Note that the spatial resolution is limited as the tip has a finite diameter of ~10 nm.

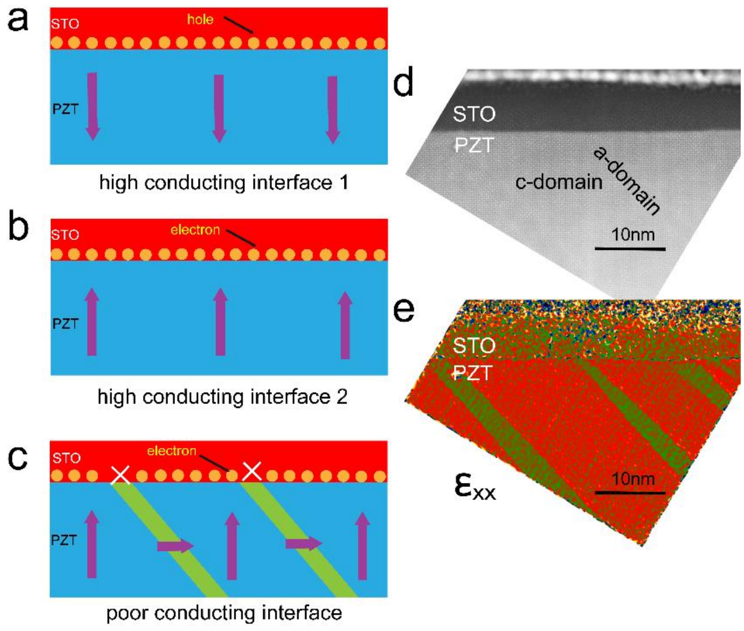

Supplementary Figure 7 | Schematics of charge distribution at STO/PZT interface. (a) Holes accumulate at STO/PZT interface when polarization is pointing away from the interface. (b) Electrons accumulate at STO/PZT interface when polarization is pointing to the interface. (c) At *a*-domain regions, no charge accumulates at STO/PZT interface, which block conducting paths and render the insulating or poor conducting state at the STO/PZT interface in a long-range. (d) HAADF image of STO/PZT interface with 90° domains. (e) Strain mapping of the image in d). The 90° domain arrays can be readily seen.

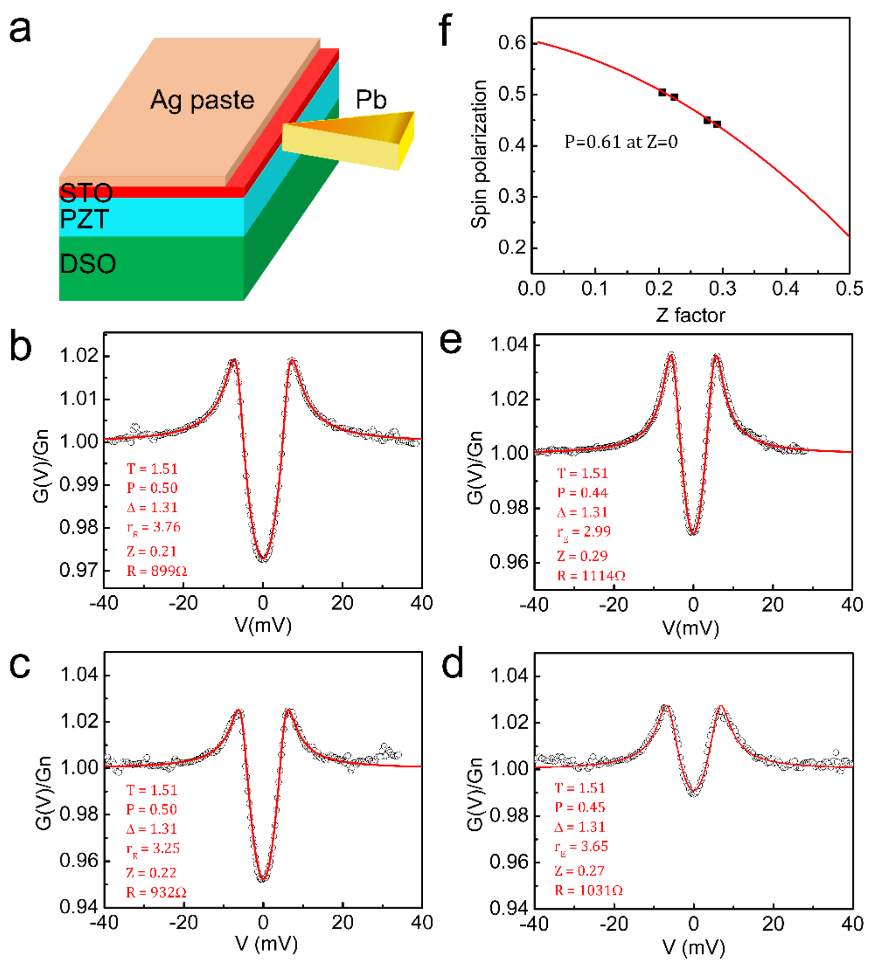

Supplementary Figure 8 | Representative Andreev spectra of contacts at 1.5 K: (a) the schematic of point contact established between Pb and the cross-section of STO/PZT/DSO. (b-e) Representative Andreev spectra (open circles) of contacts between Pb and STO/PZT interface measured at T =1.5 K with the best fits to the modified BTK model (solid curves) where the superconducting gap Δ=1.34 meV. (f) The determined spin polarization of STO/PZT interface as a function of the Z factor and the intrinsic spin polarization of STO/PZT interface.

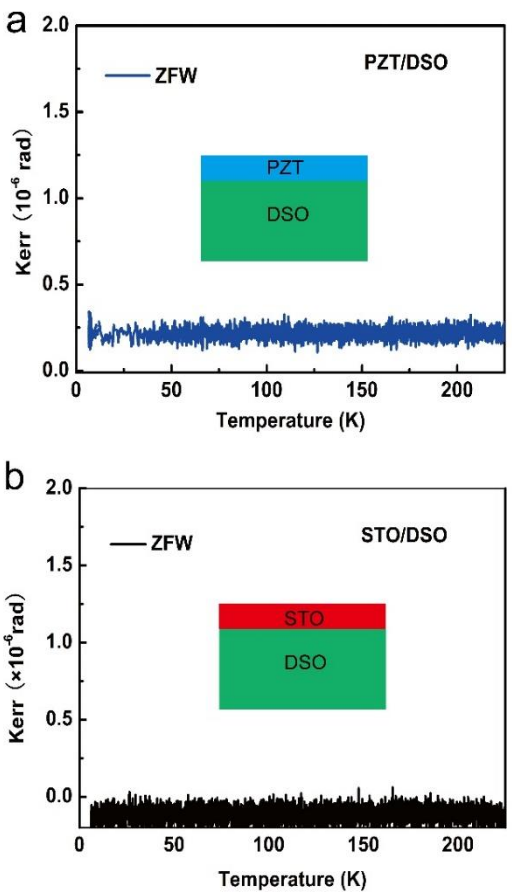

Supplementary Figure 9 | Sagnac MOKE measurements. Sagnac measurements of (50 nm) PZT on DSO (a) and (10 nm) STO on DSO (b). The samples were cooled down in zero magnetic field to 6 K. which obviously shows nonferromagnetic in both structures of PZT/DSO and STO/DSO. This data strongly suggests that the ferromagnetism only appears at the interface of STO/PZT heterostructure.

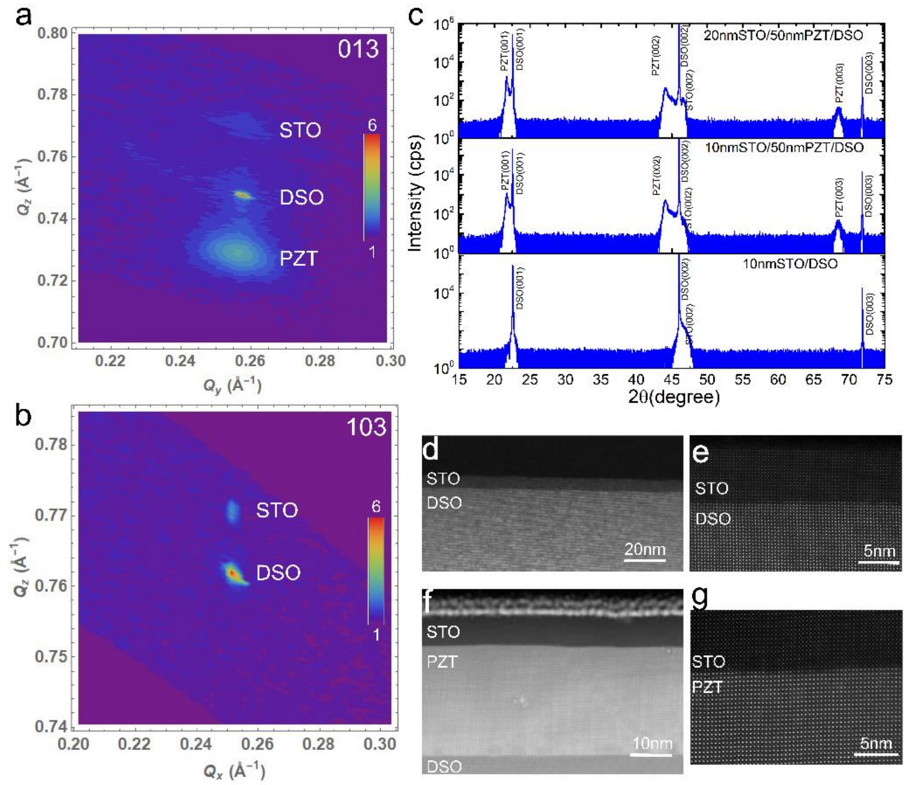

Supplementary Figure 10 | Structural characterization: (a and b) Asymmetrical reciprocal space maps confirm good epitaxial properties of STO/PZT/DSO and STO/DSO, respectively. (c) Out-of-plane HRXRD 2θ-ω scans in STO/PZT/DSO and STO/DSO heterostructures along film growth direction. HAADF STEM images at STO/DSO interface: low magnification image (d) and high magnification image (e). HAADF STEM images at STO/PZT interface: low magnification image (f) and high magnification image (g).

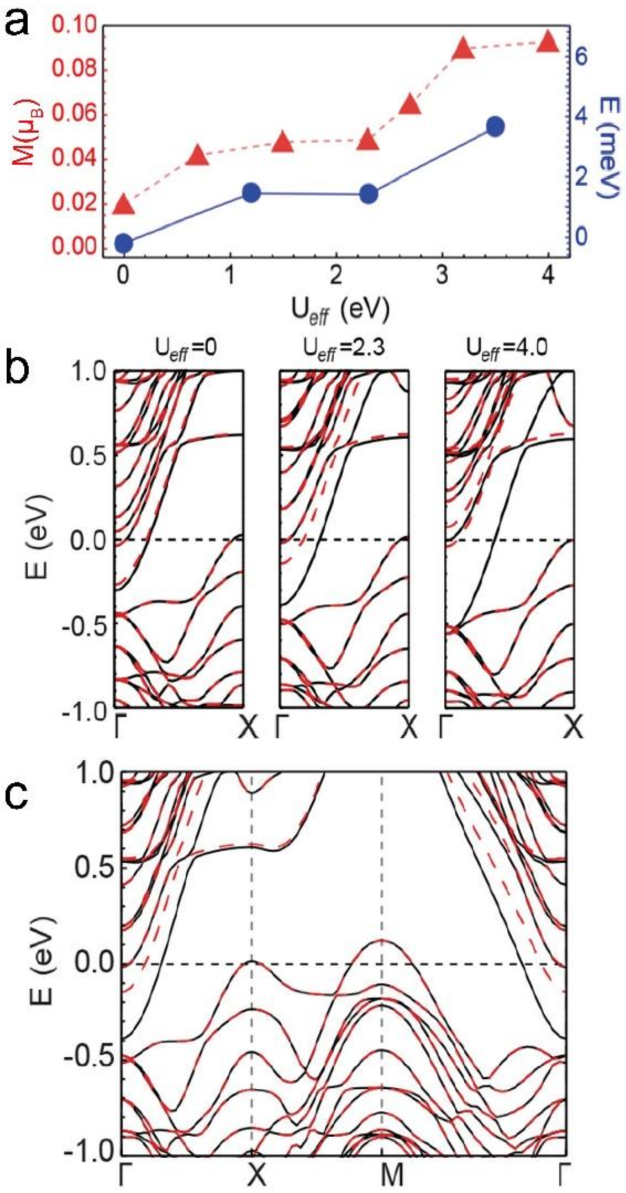

Supplementary Figure 11 | The +*U* effect on magnetization. (a) Calculated magnetization (left, red triangles) and energy gain (right, blue circles) from spin polarization of the STO/PZT heterostructure upon varying U_{eff} (*U*-J) values. (b) Calculated band structures of the STO/PZT for various U_{eff} values. (c) Calculated band structure of STO/PZT with $U_{\mathrm{eff}} = 0$ for Zr. The majority-spin (minority-spin) bands are represented by black solid (red dashed) lines.

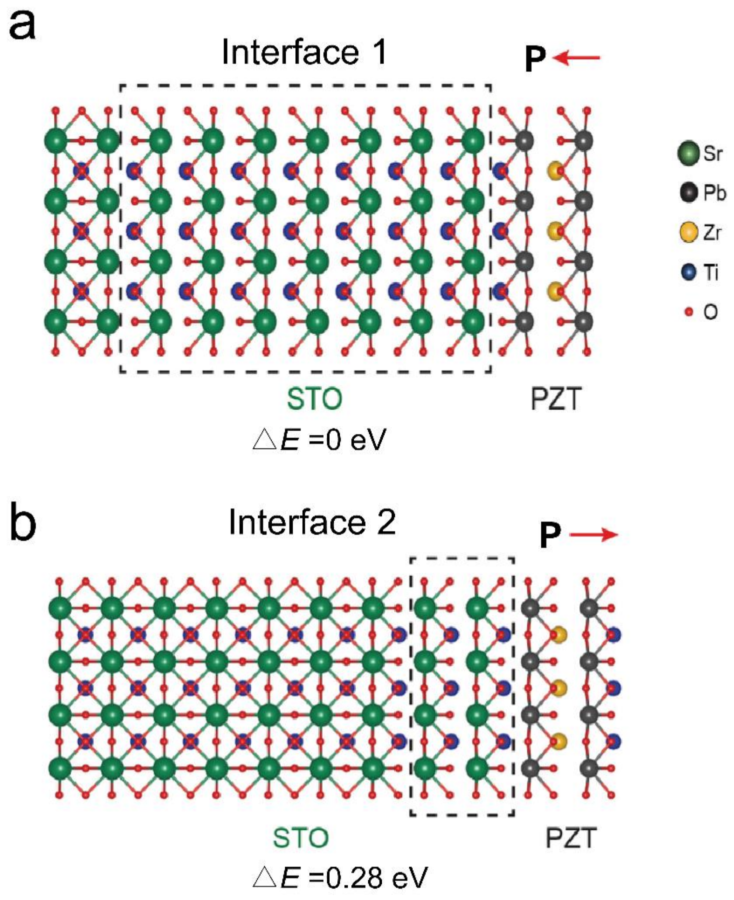

Supplementary Figure 12 | Preferential polarization direction. Atomic structure of STO/PZT heterostructures for the polarization of PZT pointing to (a) STO layers (Interface 1) and (b) the opposite direction (Interface 2). Δ*E* means the total energy difference when the energy of Interface 1 is set as 0. The chemical bonds are represented by the connected lines, with a criterion $d_{Pb\text{-}O} < 2.8$ Å and $d_{Sr\text{-}O} < 2.8$ Å. Dashed boxes highlight the STO layers that are significantly affected by the polarization of PZT.

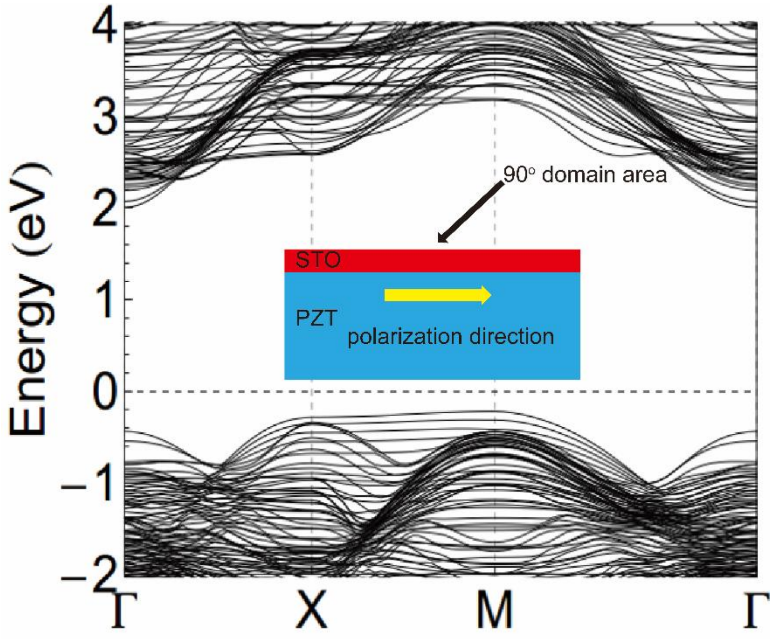

Supplementary Figure 13 | Band structure at 90° domain area. Calculated band structure at STO/PZT interface of 90° domain area, the inset shows the PZT polarization is parallel to STO/PZT interface at 90° domain area.

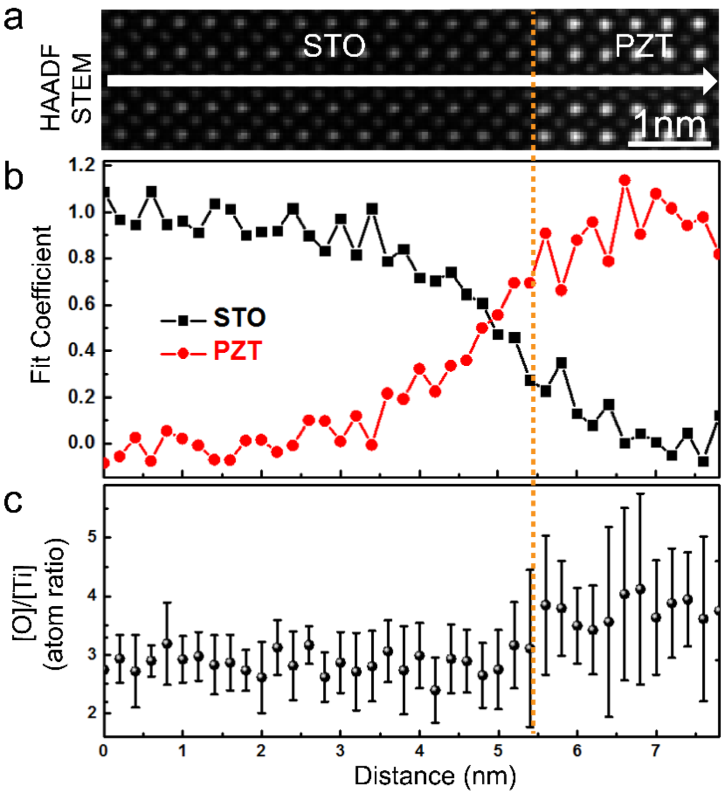

Supplementary Figure 14 | EELS analysis of oxygen at STO/PZT interface. (a) HAADF-STEM image for acquiring EELS data in STO/PZT/DSO heterostructure. Scale bar: 1 nm. (b) Fit coefficients of O *K*-edge spectra for STO and PZT, respectively, along the white arrow in (a). Using multiple least method, all O *K*-edge spectra can be well fitted by reference spectra in STO and PZT. (c) The atomic ratio of oxygen and titanium element through STO and PZT interface. Quantification process was performed in the commercial software Digital Micrograph™. The orange dashed line indicates STO/PZT interface. The average [O]/[Ti] in STO and PZT are 2.86 ± 0.19 and 3.76 ± 0.22, which is close to the stoichiometrical ratio of 3.0 and 3.75 in $SrTiO_3$ and $PbZr_{0.2}Ti_{0.8}O_3$, respectively. Near interface, we didn't find obvious oxygen deficiency in experimental error.

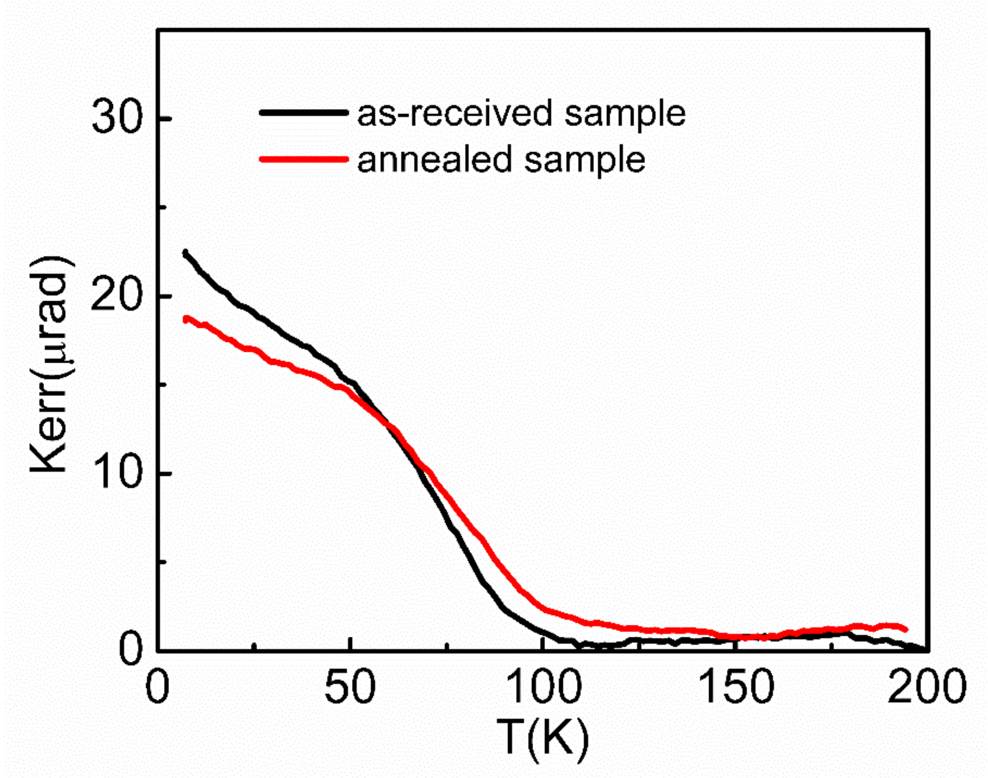

Supplementary Figure 15 | Sagnac MOKE measurements on annealed sample: as-grown STO (5 nm)/PZT/DSO sample (black curve) and post annealed in high oxygen pressure (red curve). Kerr measurements were done under 1000 Oe cool-down. The sample was annealed in an oxygen atmosphere with a super high pressure of 200 Torr

at 550 °C for 1 h to further minimize the concentration of oxygen vacancy. The O_2 pressure was always maintained both at warm up and cooling down process.

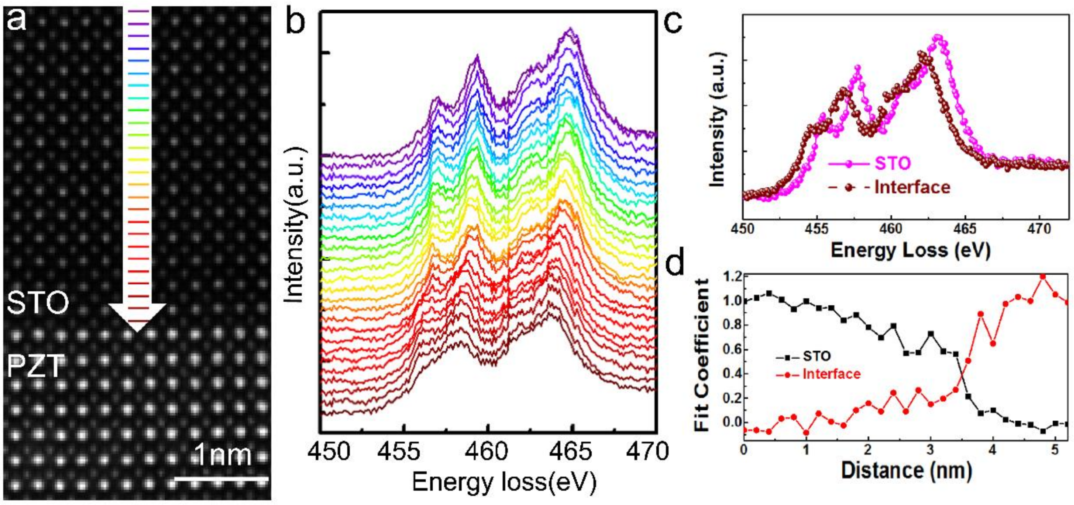

Supplementary Figure 16 | EELS analysis of Ti^{3+} and Ti^{4+} at STO/PZT interface. (a) HAADF-STEM image for acquiring EELS data in STO/PZT/DSO heterostructure. Scale bar, 1 nm. (b) EEL spectra of Ti $L_{2,3}$ edge along the white arrow in (a). Each spectrum is averaged in the direction parallel with STO/PZT interface. (c) Two typical spectra at STO and STO/PZT interface. At the interface, Ti $L_{2,3}$ edge has a visible shift to lower energy value, compared with those in the bulk of STO, indicating the existence of Ti^{3+} at the interface. (d) Fit coefficients of Ti spectra for STO and STO/PZT interface. To separate the contributions of Ti^{4+} in STO and Ti^{3+} at interface, we used multiple least square (MLS) methods in the following equation to fit each experimental spectrum in each pixel by three reference spectra, $S(E) = a_1R_1(E) + a_2R_2(E) + a_3R_3(E) + \chi(E)$, where E is energy loss, $S(E)$ is the experimental EEL spectra from each pixel, a_1, a_2 and a_3 are fit coefficients (spectral weight) of Ti spectra at STO, interface, and PZT, $R_1(E)$, $R_2(E)$ and $R_3(E)$ are reference spectra of STO, interface, and PZT from (c), and $\chi(E)$ is residual spectra. The fitting process was performed in the commercial software Digital Micrograph™. The orange dashed line indicates STO/PZT interface. It is intriguing

that Ti^{3+} is only appeared in STO film and in the range of 2.5 nm away from STO/PZT interface.

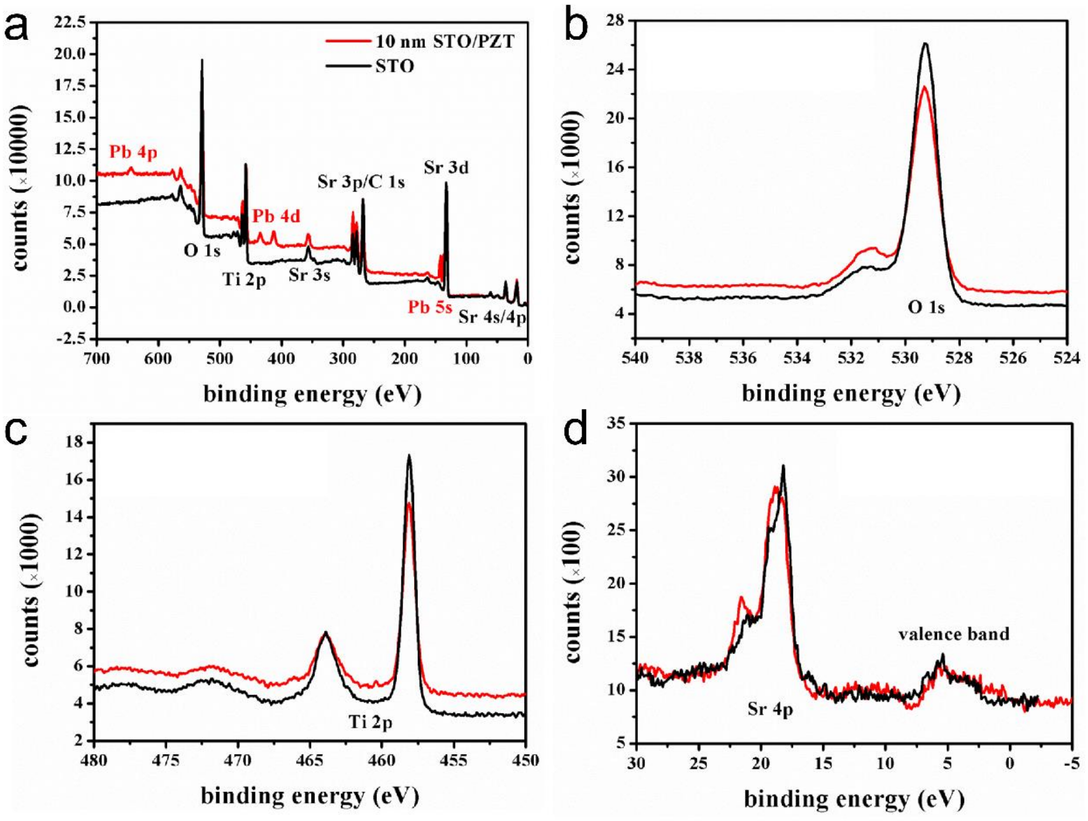

Supplementary Figure 17 | X-ray photoemission spectroscopy measurements. (a) the spectra of the STO (10nm) /PZT film as well as pure STO substrate as a reference. (b and c) Fine X-ray induced photoelectron core level spectra of O 1s and Ti 2p emissions. (d) The valence band electronic structures. The penetration depth is less than 5 nm.

Supplementary Notes

Supplementary Note 1: XRD analysis

Supplementary Figure 1a shows that there are only (00L) peaks observed in the out-of-plane 2θ-ω scan for all the STO/PZT/DSO films, confirming that the films are

single crystalline and grown with the (00L) parallel to the sample surface, as expected for epitaxial growth.

Supplementary Figure 1 b and c show the views of the symmetric DSO (002) and asymmetric (013) reflections, respectively, in which the peaks of the STO, PZT, and DSO are separate and pronounced. From symmetrical DSO (002) RSM, the peaks for thin layers of STO and PZT are symmetric about $Q_x = 0$ indicates there is no tilt or curvature on the film. Two weaker scattering wings suggest the existence of PZT a-domains. From the asymmetrical DSO (013) RSM, the centers of film peaks STO (013), PZT (013) and substrate peak DSO (013) are well aligned at $Q_x = 0.257A^{-1}$. This indicates that the film was fully epitaxially grown on the substrate and the lattice of the film is strained in a manner such that the in-plane lattice constant of the epitaxial film and that of the substrate match, at least along the Q_x direction.

Supplementary Figure 2 also demonstrates the growth of high-quality epitaxial and single crystalline of STO/PZT/SRO/DSO films.

Supplementary Note 2: XPS analysis

Supplementary Figure 17a shows the survey spectra of the as-received STO/PZT film as well as pure STO substrate as a reference. Evidently, only emissions from Sr, Pb, Ti, O and C are found in the STO/PZT/film and no other element could be observed. The presence of C 1s emission is due to the surface adventitious carbon. Thus, the possibility of chemical doping during the growth can be completely ruled out.

Fine X-ray induced photoelectron core level spectra of Ti 2p and O 1s emissions are shown in Supplementary Figure 17b and c. Binding energies are checked carefully with respect to the C 1s peak of surface carbon (~284.8 eV) for all the samples and no charge induced shift was found. The core level binding energies of Ti $2p_{3/2}$ (~458.1 eV) and O 1s (~529.3 eV) emissions are obtained and they are in excellent agreement with reported results from pure STO. This good consistency suggests that there is no core level binding energy shifts within STO thin films. Furthermore, note that the Ti 2p emission peaks of STO/PZT are identical to that of referencing STO. Therefore, it can be

concluded that a mixture of Ti^{3+}/Ti^{4+} valence state at room temperature is very small in the bulk of STO layer and beyond the detection limit of the instrument, which is consistent with our EELS data. The valence band electronic structures of STO layers were investigated as well and the results are displayed in Supplementary Figure 17d, which is almost identical to the standard STO.
