# Supplementary material for: Discovery of a magnetic conductive interface in PbZr0.2Ti0.8O3 /SrTiO3 heterostructures
Source: Nat Commun. 2018 Feb 15;9:685. doi: 10.1038/s41467-018-02914-9 (PMC5814552; doi:10.1038/s41467-018-02914-9)
Supplement: Supplementary file 2 — Description of Additional Supplementary Files [file 41467_2018_2914_MOESM2_ESM.pdf]

Description of Additional Supplementary Files

File Name: Supplementary Movie 1
Description: **Domain switching behaviors in PZT/SRO/DSO.** Domain evolution under linear 0→9 V observed by *in-situ* TEM. The time duration of the applied voltage is 3 s. The applied voltage starts at the beginning of the video.

File Name: Supplementary Movie 2
Description: **Domain switching behaviors in STO/PZT/SRO/DSO.** Domain evolution under linear 0→–6 V observed by *in-situ* TEM. The time duration of the applied voltage is 20 s. The applied voltage starts at the beginning of the video.
